# How to coach student professional development during times of challenges and uncertainties

**DOI:** 10.1186/s12909-023-04588-4

**Published:** 2023-08-22

**Authors:** Annelies E. van Ede, Roy J. M. Claessen, Merel van Gils, Wim J. M. J. Gorgels, Rob P. B. Reuzel, Annemieke G. J. M. Smeets, Petra J. M. van Gurp

**Affiliations:** 1 Department of Rheumatology, Radboud University Nijmegen Medical Centre NL, Nijmegen, Netherlands; 2 Department of Internal Medicine, Radboud University Nijmegen Medical Centre NL, Nijmegen, Netherlands; 3 Radboud Health Academy, Radboud University Nijmegen Medical Centre NL, Nijmegen, Netherlands; 4 Department of Primary and Community Care, Radboud University Nijmegen Medical Centre NL, Nijmegen, Netherlands; 5 Department of Health Evidence, Radboud University Nijmegen Medical Centre NL, Nijmegen, Netherlands; 6 Department of Pathology, Radboud University Nijmegen Medical Centre NL, Nijmegen, Netherlands

**Keywords:** Professionalism, Curriculum, Lifelong learner, Self-directedness, Adaptive competence, Identity formation, Practice-based learning

## Abstract

**Background:**

What we teach our (bio)medical students today may differ from the future context under which they will operate as health professionals. This shifting and highly demanding profession requires that we equip these students with adaptive competencies for their future careers. We aimed to develop a framework to promote and facilitate professional development from day one, guided by self-awareness and self-directed learning.

**Approach:**

Based on self-directed, transformative and experiential learning, patient involvement and teamwork, we developed a 3-year longitudinal personal-professional development (LPPD) program in the (bio)medical sciences undergraduate curriculum to stimulate self-driven professional development in a variable context. Through group meetings and individual coach consultations, students address topics such as self-awareness, self-directed and lifelong learning, collaboration, well-being and resilience. To drive learning students receive extensive narrative feedback on an essay assignment.

**Evaluation:**

Experiences and outcomes were evaluated with questionnaires and in-depth interviews. Students and coaches value personal and professional development in a safe learning environment that encourages self-exploration, diversity and connection. Over time, students show more self-awareness and self-directedness and increasingly apply trained skills, resulting in professional identity formation. Students need more clarification to understand the concept of assessment as learning.

**Implications:**

With the generic content of a longitudinal program embedded in a meaningful environment, the personal and professional development of students can be facilitated and stimulated to face future challenges. When translating to other curricula, we suggest considering the complexity of professional development and the time expenditure needed for students to explore, experiment and practice. An early start and thorough integration are recommended.

## Background

Healthcare and research environments are changing rapidly [1]. To prepare for future challenges, Frenk et al. proposed focusing on the so-called T-shaped professional, incorporating ‘breadth (generic professional competencies) across fields and depth (expertise knowledge and skills) in primary fields.’ In addition, universities have noticed an increase in students’ mental issues and a loss of well-being that needs appropriately addressed [2, 3].

For this endeavor, an adaptation of the current (bio)medical curricula seems inevitable [1]. In addition to the more traditional knowledge transfer from teachers, our students need encouragement to become self-directed, lifelong learning, flexible, adaptive and resilient professionals. The self-determination theory of Ryan and Deci, in which the importance of a self-directed role and attentional motivation, positive identity-formation and well-being are emphasized, supports the need to revise the curricula [4, 5]. A major question regarding (bio)medical curricula involves the best means to adequately prepare students for their future challenges in the healthcare working environment. Although there is ample focus in the literature on education in professionalism, it mainly concerns defined, targeted learning activities, the identification of unprofessional behavior, or mentoring roles [6, 7]. The importance of mentorship in guiding professional development is well recognized, as is the value of the mentorship in itself [8, 9]. Uncontrolled informal learning in the area of professional performance and mentoring is still often executed empirically using ad hoc methods and approaches, which results in an undesirable range of consistency and effectiveness [10]. Although they are needed, there is a gap in the literature concerning more elaborate and effective programs, including a framework and clear toolkits for the promotion of professional development, that covers and integrates the entire undergraduate curriculum.

In this paper, we describe the background, content and experiences of both students and teachers within a new, multidisciplinary (faculty, students, educationalists) participatory cyclic design process that was developed and implemented in a three-year undergraduate educational longitudinal personal-professional development (LPPD) framework.

## Approach

Our new LPPD program is designed as a part of the revised curriculum Medicine and (Bio)Medical Sciences that was developed at the Radboud University in Nijmegen, the Netherlands, and started in 2015. The 4 leading educational principles of the new curriculum include an active, collaborating and self-directed role of the students, in a practice based and patient centered environment. LPPD is implemented as one of the five basic longitudinal educational courses in the undergraduate program and includes 14 EC of the total of 180 EC.

**Fig. 1:**
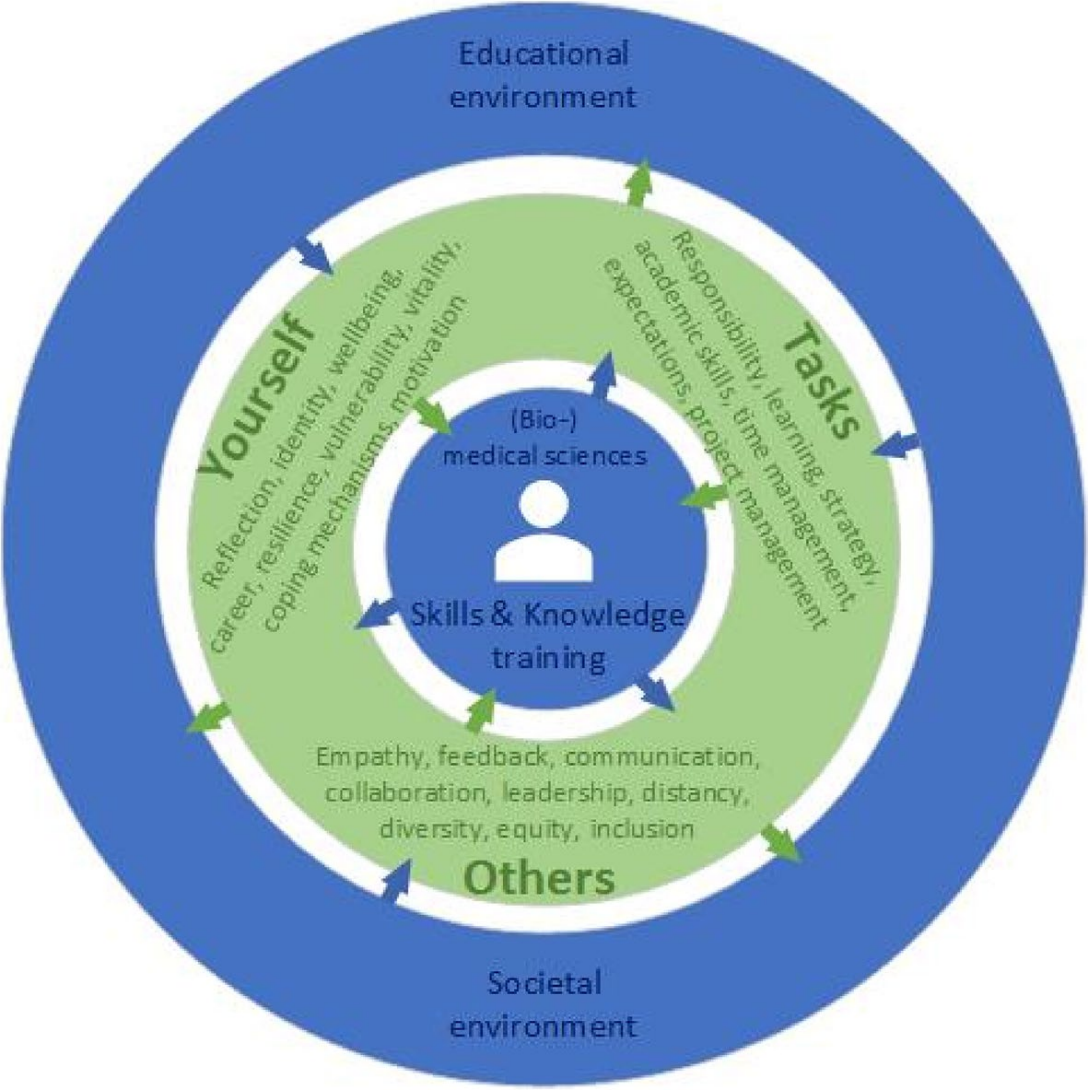
A (bio)medical science student in our curriculum attends LPPD (in green), which is focused on three domains (taking care of one’s self, others and daily tasks) and is closely connected to the educational and societal environment of the student and the core of the (bio)medical curriculum (in blue). This close connection enables the exchange of experiences and input for professional development and the possibility of developing new skills throughout the entire curriculum (indicated by arrows)

LPPD aims at encouraging personal and professional self-awareness and identity formation (in relation to oneself, to task performance, and to teamwork in a variable and increasingly complex context, as shown in Fig. 1) as expressed through professional attitude and behavior, rather than the detection of unprofessional behavior, the leveraging of corrective measures and the exclusion of students exhibiting bad behavior [11–13].

The LPPD design is based on the educational theories of self-determination and constructivism, and experiential and transformative learning are embedded in a curriculum where students meet patients on day one [4, 5, 11–19].

### Program – content and learning activities

**Fig. 2:**
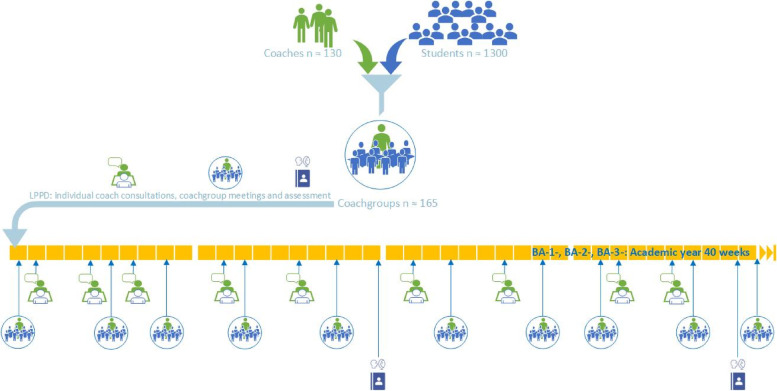
Layout of the LPPD, coach group sessions (*n* = 10), intertwined with individual coach–student meetings (*n* = 9). Every calendar academic year, students in their first study year (BA-1-), second year (BA-2-) and third year (BA-3-) participate in the LPPD course. Every year, students and coaches enroll in a new coaching group. The structure is similar across BA-1-, BA-2- and BA-3-, the content is diverse, and students progress through a context with increasing levels of complexity

The LPPD framework consists of a 3-year longitudinal, mandatory program. Each academic year, all students are assigned to (new) coach groups of 8–9 students each and a new teacher who has well-developed coach qualities (the so called ‘coach’). The program includes scheduled 30-min individual consultations with the coach as well as 2-h coached group sessions during each academic year, and both activities have preparation learning activities to be completed in advance of the meetings (Fig. 2). Coaches guide and stimulate student development toward self-direction and self-confidence [20]. Coaches have the ability to encourage students’ self-discovery journeys to enhance professional development and unlock their potential. Coaches effectively guide group meetings, aimed at promotion of peer-assisted learning and awareness of diversity of students’ perspectives, needs and experiences. The coaching role should not be confused with the activities of study counselors. Students consult study counselors on demand in the case of study delays or major (health) issues that impact their studies. Coaches for LPPD are selected from (bio)medical teachers within the curriculum, because of their endorsement of the importance of professional development for students and their connection with the students’ intended future field of work [9]. All coaches participate in a training program and workshops [9].

When a new coaching group is formed, students need structure and clearly defined expectations. Over the year, to enable just-in-time learning, students are invited to take increasing ownership of group sessions and compose a portion of the agenda and the matching learning activities. A toolbox with a variety of learning activities is available for coaches and students to promote autonomy, self-directed learning and attention to diversity. Students and coaches themselves, as well as educators, introduce original activity formats to inspire and encourage students in their learning and professional development. Activities with new formats designed by students and their coaches are collected by the steering committee. In this way, students, coaches, educators and steering committee members, cocreate a toolbox that is continuously replenished (Table 1). The role of coaches is pivotal in making the content applicable to students and aligned with individual development.

**Table 1:** Overview of LPPD (themes and content). Learning skills and general professional skills are regularly and repetitively addressed with emphasis on learning as an active, contextualized, practice-based, lifelong process of constructing knowledge and skills from the psychosocial environment and personal experiences. Menu and toolbox: Because one size does not fit all, we provide the students and teachers with a menu of a variety of learning activities that can be applied to various topics within the LPPD. A toolbox is included with which to select the most suitable forms of student engagement

***LPPD cornerstones:***
• A compulsory program for every student, intertwined throughout all courses in the curriculum, guided by a coach and aimed at personal and professional identity formation and performance, with room for diversity, that connects to society and healthcare
• Alternating prepared group sessions (2-hours) and individual meetings (30 minutes each) with a coach, spread throughout the academic year and including a final assignment.
• Incorporates the educational theories of self-determination and constructivism, experiential and transformative learning, and learning as an active, contextualized, practice-based, lifelong process of constructing knowledge and skills based on psychosocial environment and personal experiences.
***Themes and content of the LPPD program***
• Landing at the university (semester 1): Discovering oneself, including the professional that one could and would like to become and the requirements for achieving that goal, constituting the beginning of professional development and lifelong learning, including learning strategies, critical thinking, and creativity)
• Collaboration (semester 2): As a (bio)medical student, one cannot study without patients, fellow students and teachers. It is (semester 2): As a (bio)medical student, one cannot study without patients, fellow students and teachers. It is important for one to rely on, or work toward, good cooperation. Team building and team work, feedback, and Belbin roles are all explored.
• Personal leadership (semester 3): Leadership necessitates discovering and developing oneself. Self-evaluation requires taking control of one’s own development and functioning. Self-direction involves looking in the mirror, thinking about best practices (reflecting) and making learning goals concrete. Reflection skills, self-awareness, and identity formation are key.
• Self-exploration (semester4): Lifelong learning, wellness, vitality, and vocational orientation contribute to the development of self-knowledge. A personal needs assessment is applied to assist you in knowing who you are, what your qualities are and what pitfalls you need to learn to avoid.
• Connection with the society (semester 5): Students come to understand the societal perspective and impact. To broaden and deepen the future roles of students in health-advocacy, with attention to diversity and inclusion, crossing boundaries and ethical considerations, social interaction and engagement, integrity, professional standards and values are explored and identified.
• Back to the future (semester 6): To develop an overall career perspective, students deal with the choices to be made, prepare for internships, and reflect and plan with motivation, pride and trust.
***Skills training (repetitive learning in an increasingly complex context)***
• Academic skills, including critical thinking, gaining insight, developing understanding, and writing and presenting in an academic context
• Reflection skills
• Feedback: ask, give and receive
• Feed up and feed forward
• Stress reduction, vitality and resilience techniques
• Group dynamics and roles, how to select group members to work with
• Time and project management, planning organization, allocation of roles and tasks
• Communication and cooperation skills in (hierarchic) relationships
• The exploration of personal talents and pitfalls, and how to use this knowledge
• Self-directed and peer-assisted lifelong learning
• Feasible goalsetting
• Mindset and mind mapping
• Creating and maintaining a safe learning environment
• Maintaining one’s own well-being, flexibility and resilience, as well as the development of empowerment
• Developing an awareness of diversity and inclusivity
***Toolbox of learning activities in connection with learning concepts (LC), facilitated by the coaches***
• Theory: literature, e-learnings, illustrating interviews, illustrating movies (LC: self-directed learning, just-in-time learning)
• Reflection and discussion: both individually with a coach or in small group settings and through group debates on actualities, patient stories, film fragments, simulation games, reflection reports (LC: peer-assisted learning, practice-based learning, learning by doing, transformative learning)
• Skills training: practical assignments (in relation to other learning activities in the curriculum), workshops, self-evaluations, individual gaming, live gaming with a group, group debate, and student story-telling (LC: peer assisted learning, practice based learning)
• Coach-consultation sessions centered on humble inquiry (LC: experiential and transformative learning)
• Working groups with student input (LC: just in time learning, based on actual experiences, peer assisted learning)
• Questionnaires, self-evaluation tests and assessments (LC: transformative learning)
• Actualities: societal engagement (LC: constructivism, practice-based learning, boundary crossing)
***Student toolkit***
• Structured reflection guideline (pocket size) and self-evaluation template
• Strength tests
• Feedback and feed forward tools
• E-portfolio with personal stories, experiences and insights
• Time management; project management tools
• Safety in a learning environment

The LPPD program is organized into six semesters, each with an overarching theme that connects with the other topics. An overview of the themes and learning activities of the 6 consecutive semesters in the LPPD are listed and explained in Table 1. Learning activities are aimed at encouraging a personally formative contribution from the student, providing both a normative framework (aimed at the standards, rules, and social context of the outside world) and a restorative contribution (aimed at maintaining interest and intrinsic motivation). Coaches continuously ask students to engage in exploration. Students are encouraged to evaluate effectiveness of their behavior to grow as professionals. In case of observed incorrect behavior coaches address this observation with the student. If more severe mediation is necessary, then it is made available to supplement the LPPD program.

Throughout the program, ample attention is given to self-reflection, personal well-being and resilience [21, 22]. In dialog with coaches and peers and through assignments meant to explore social and emotional skills, students are guided in improving their psychological flexibility and thus their resilience and vitality [3, 20, 23]. Developing as a professional implies becoming aware of one’s personal strengths and weaknesses, the personal values that guide behavior and the underlying dynamics that affect one’s behavior. Understanding one’s own core values helps the student learn how to respond effectively to situations that might be encountered as a professional. Moreover, it helps one stay true to themselves in challenging situations. Students explore and experiment with alternative behavior appropriate to the variable context on the basis of self-reflection and feedback. Taking care of one’s own physical and mental health, preserving a balanced healthy standard of well-being, and assuring an understanding of the limits of one’s own competencies and how to act accordingly are important elements that contribute to a successful professional career.

Within the LPPD program, students learn generic skills (following Miller’s pyramid level know and level knows how) [24]. The close connection of LPPD with other learning activities within the (bio)medical sciences curriculum facilitates a meaningful environment within which students can apply their knowledge and trained skills. Conversely, the overall learning environment and learning across the curriculum is also the context from which a student determines what areas need to be developed and expanded. Students learning professionalism can acquire practice through their major assignments and through traineeships in other parts of the curriculum, where students are observed for several weeks or months (Miller’s pyramid level shows) [24]. Overall, the LPPD program was developed to facilitate a connection between patient care, education, science and society. In Table 2, the relation of LPPD to the rest of the (bio)medical sciences curriculum is illustrated.

**Table 2:** Opportunities for alignment of LPPD with other areas of the curriculum. These examples show the opportunity for beneficial learning outcomes within the entire curriculum. The students use competencies learned and trained through LPPD to strengthen the effectiveness of the learning process in other parts of the curriculum

***LPPD: opportunities for alignment with other activities in the (bio)medical curriculum enriches learning outcomes***
**In the context of the science program:** students conduct a scientific project in collaboration with 3–6 of their peers. The roles, task and project management, and difficulties that they face during this collaboration and their academic skills training are discussed in coaching groups. They learn to respect the other and to commit to diversity in both people and process.
**In the context of practical skills training:** students learn what it means to do a physical examination, how it feels to examine patients (empathy and sympathy), how to begin and maintain a relationship, and how to navigate privacy issues and uncertainties, etc. In the coaching groups, they discuss how to relate to these issues and how to reflect on these experiences for personal and professional identity formation.
**In the context of the mechanisms of diseases:** students attend lectures and working groups. Within the coaching group, students encourage each other to explore learning strategies. They recognize the benefits of peer-assisted learning and learning communities in motivating them to participate in learning activities. Students discuss critical thinking, how to consult an expert, the power of peer-assisted learning, and how to identify a suitable learning strategy.
**In the context of early patient contacts:** students explore the patient experiences in the healthcare system. They explore the individual person behind the patient and the societal perspective on healthcare. In the coaching groups, students share their experiences with these real patients to facilitate transformative learning.
**In the context of a chosen minor program:** Self-determination is encouraged through the coaching groups. Students verbalize the stress generated by the making of choices, and they get to work on personal leadership in small group teaching.

### Program – evaluation of learning outcomes

Students manage their professional development through an e-portfolio in which written reflections, self-evaluation reports, and feedback documents are curated. Additionally, twice a year (per semester), with an essay assignment provided with extensive narrative feedback, we shaped the learning concept of ‘assessment as learning’. This assessment is accessible for all students without external mediation in the event of persistent incorrect behavior. In a personal evaluation essay on their professional development, students apply knowledge and trained skills through an illustration of their growth and their uncertainties through reflections and multisource feedback. Students formulate individual learning goals in advance for each semester that align with their self-evaluation and reflect on the learning goals of the preceding semester. The essays are rated and provided with extensive narrative feedback and feed forward by assessing coaches. In addition to coaching the students, all coaches also play a role in assessing the students that they do not coach themselves.

We opted for a written essay assignment, as describing the progression of professional development shows a higher level of professional authenticity than only describing applications in an exam [25]. Reflecting on thoughts, feelings and personal development provides an extra, deeper dimension of learning [26]. Writing expands the use of higher level thinking through repetitive recall, the exploration and understanding of content, the application of critical thinking skills, social skills and an awareness of societal context, and the internalization of learned concepts [26]. The ‘assessment as learning’ assignment appeals to meta-cognitive skills, and it includes questions such as what is the purpose of learning concepts of professionalism and skills? Do I understand these concepts, and have I accomplished the goals I set for myself?

## Evaluation

### Data collection and analysis

Over 1300 students spread over bachelor’s years one, two and three, and approximately 130 coaches participate in the yearly LPPD program each academic year. Within the participatory cyclic design process of LPPD, we collected experiences yearly (2016–2021) using questionnaires for both students and coaches. In 2020–2021, semi-structured and in-depth focus group interviews were conducted with both students and coaches to obtain further insight into the experiences of students and coaches, and for triangulation.

The questionnaires consist of 23 Likert scale statements and three open-ended questions, and were developed in consultation with faculty and students. Student and coach questionnaires were completed voluntarily and analyzed anonymously. From all available evaluation data, we selected the paired student-coach data (2020–2021). These data show the experiences after a three-year development cycle (2015–2018) and the first major improvement actions (2018–2021), and illustrate the representative outcomes and effects of LPPD from student and coach perspectives. The data from questionnaires, which was based on a quantitative Likert scale (1–6), were inputted into an Excel spreadsheet for analysis.

The concept interview guide was designed with input of faculty, coaches and students and finalized by AE and PG. The semi-structured focus group interviews were conducted by an independent researcher. Students and coaches were interviewed separately to ensure a comfortable and open atmosphere to deepen discussions. All interviews were audio-recorded after obtaining informed consent from the participants. The interviews were transcribed and coded, and an inductive thematic content analysis was performed using ATLAS.ti. Representative quotations illustrating gained insights from the interviews have been translated into English.

### Ethics approval

This study was approved by the Research Medical Ethics Committee (CMO) of the Radboud University Medical Centre (case number: 2020_6518). Students and coaches were informed about the use of evaluation data. Informed consent was obtained from all participants. Data were analyzed anonymously.

## Results

### Student and coach responses

A total of 1356 students were invited to complete a questionnaire in the 2020–2021 academic year, of which 698 (51%) were completed (65% for bachelor’s year 1; 71% for bachelor’s year 2 and 19% for bachelor’s year 3). All students were invited to participate in semi-structured in-depth focus group interviews. From all students that applied, we randomly selected students from bachelor years one, two and three, male and female, to participate in the interviews until data saturation was achieved after seven focus groups and a total of 35 students.

A total of 127 coaches were invited to complete a questionnaire in the 2020–2021 academic year, of which 81 (64%) were returned completed (65% of bachelor’s year 1, 63% of bachelor’s year 2, and 65% of bachelor’s year). From all coaches that applied, we randomly selected coaches from bachelor years one, two and three, male and female, to participate in the interviews, until data saturation was achieved after 4 focus groups and a total of 20 coaches.

**Fig. 3:**
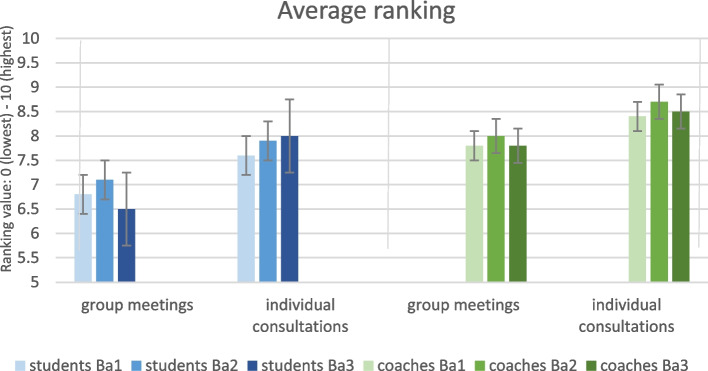
LPPD: Results of student and coach evaluations (questionnaires, academic year 2020–2021) showing levels of appreciation for the content of LPPD

**Fig. 4:**
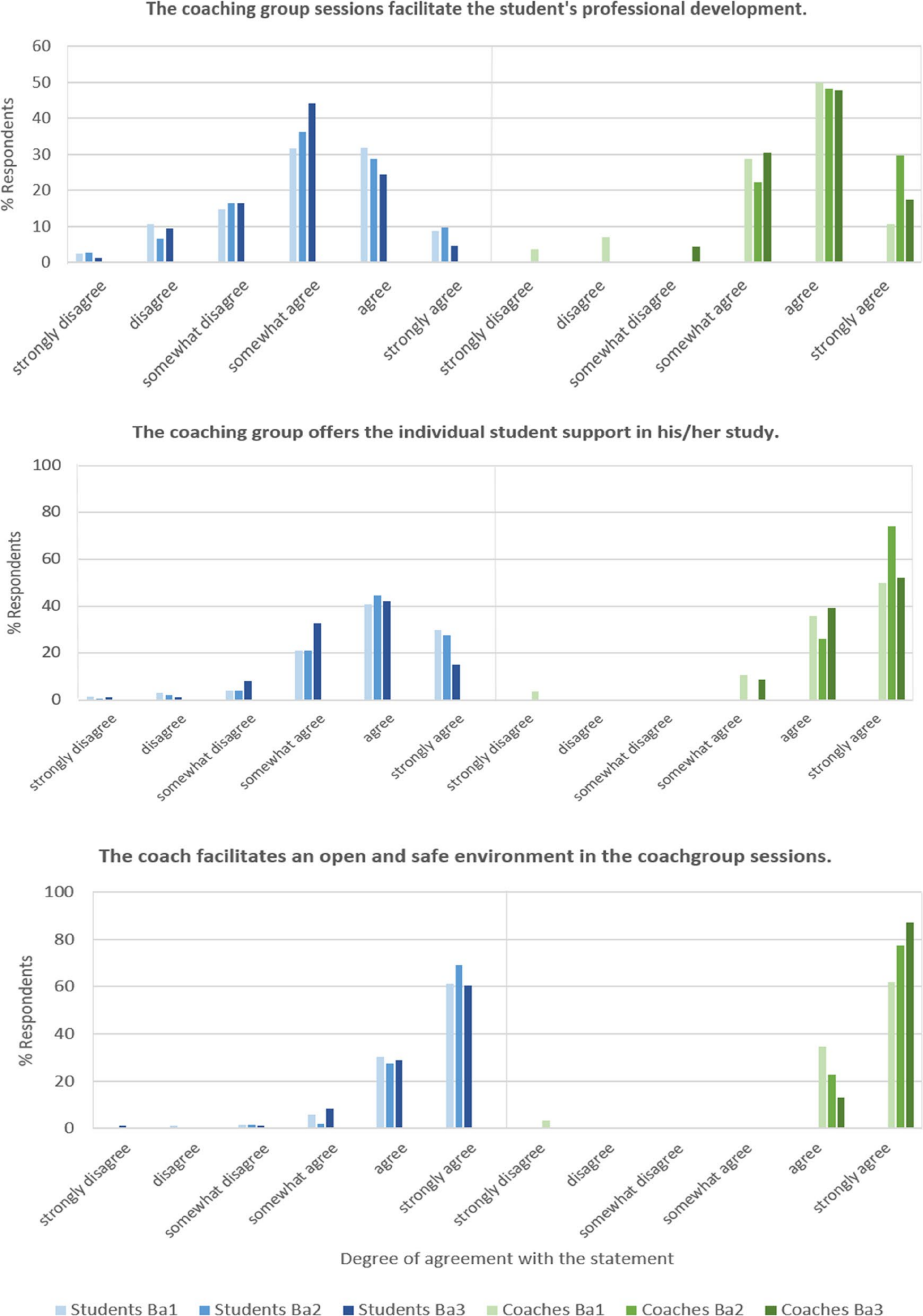
LPPD: Results of student and coach evaluations (questionnaires, academic year 2020–2021). This graphic shows the degree of agreement with 3 statements concerning the contribution of the coach group (sessions) to the learning process of the student

**Fig. 5:**
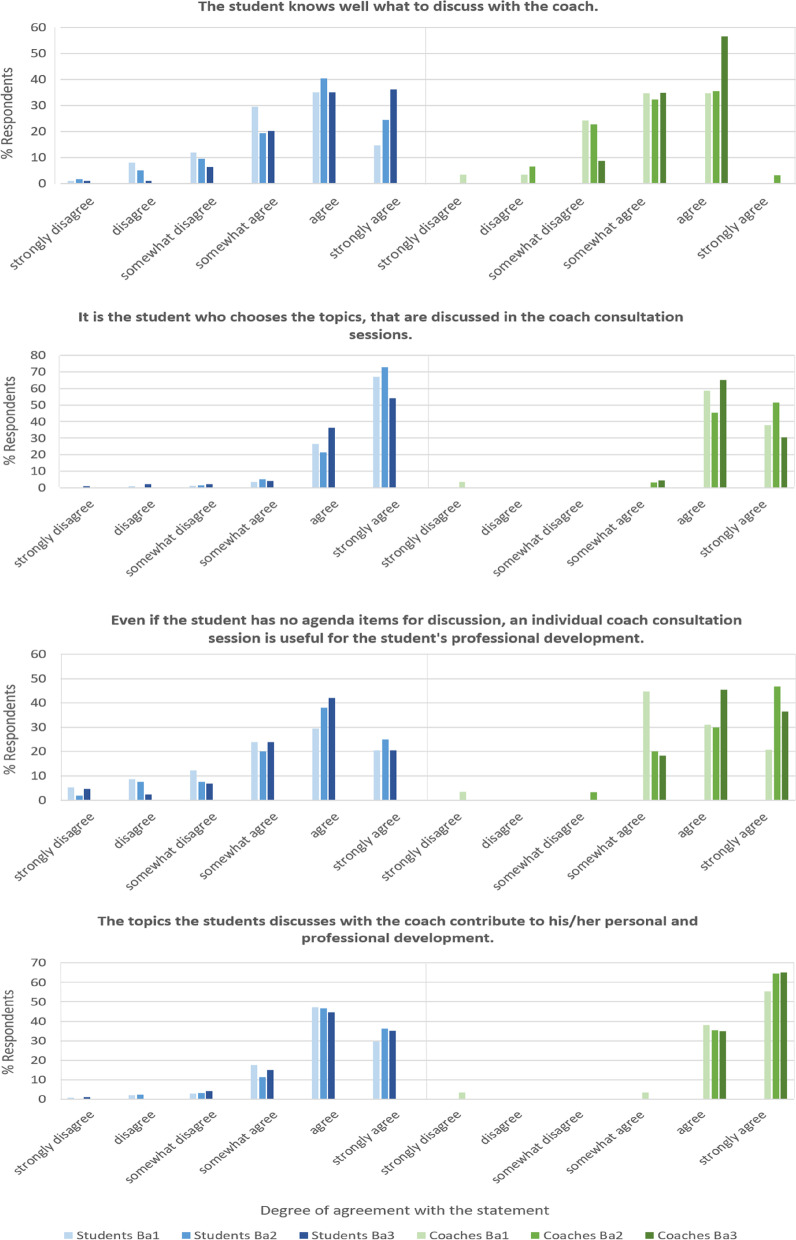
LPPD: results of student and coach evaluations (questionnaires, academic year 2020–2021). This graphic shows the degree of agreement with 4 statements concerning experiences of individual consultations, being a learning activity in LPPD

**Fig. 6:**
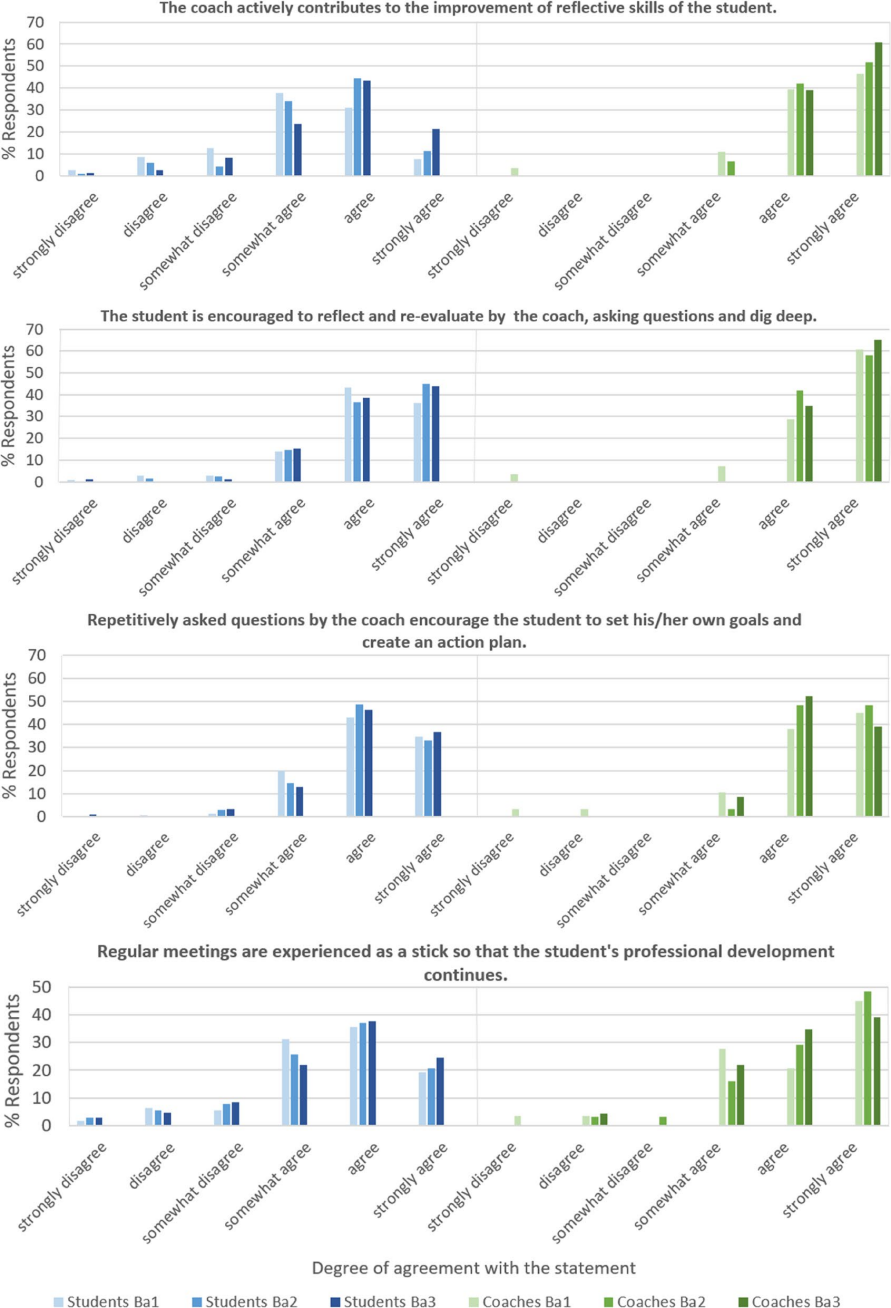
LPPD: results of student and coach evaluations (questionnaires, academic year 2020–2021). This graphic shows the degree of agreement with 4 statements concerning professional skills development, being stimulated by coaching

To give the reader a proof of our concept, we present statements, that are most illustrative and provide insight into students’ and coaches’ experiences with LPPD. The complete questionnaires are available from the corresponding author upon request. In Figs. 3, 4, 5 and 6 we show the paired results of students’ and coaches’ responses on several topics within LPPD. Figure 3 shows the ranking of the learning activities within LPPD. The rating for LPPD group sessions is similar to other programs (i.e. anatomy, practical medical skills training) within the curriculum. Individual consultations are rated above average, and valued more than group meetings by students. Overall, coaches rank LPPD, both group sessions and individual consultations significantly higher than students (*p* < 0.01).

In Fig. 4 we present the results of statements concerning experiences with group meetings. Figure 5 represents the data of experiences with individual consultations as a learning activity, and Fig. 6 represents professional skills development as stimulated by coaching. Finally, in Tables 3 and 4 quotations (Q) from students’ (QS) and coaches’ (QC) interviews respectively, illustrate the LPPD program experiences, learning outcomes, and assessment.

**Table 3:** Students' quotes concerning the LPPD: program (experiences and learning outcomes) and assessment (interview data 2020–2021)

***LPPD—program***
***Experiences***
*S1: ‘Within the individual sessions with my coach, I became aware of what I (sometimes unconsciously) learned from my experiences within other parts of the curriculum. I learned to think about why things happen the way they happen.’*
*S2: ‘From vague and useless to awareness of usefulness. I have developed from unconsciously incompetent to consciously competent in the area of professional student attitude and behavior. I feel the need to develop even further. My peers in my coaching group were my colleagues in this sometimes uncomfortable but finally inspiring process.’*
*S3: ‘I must admit that at the beginning, the course on professional development annoyed me very much because I thought it was not useful for me. Looking back now, I may have learned the most from this course in the whole curriculum. I know much better who I am, how my actions and behavior come about, how I can change them and what I need to do so. So my new learning goal is: 'Not to immediately resist something I do not think is useful, but being open to other views.’*
*S4: ‘I've figured out myself, at least in the last few years, which aspect of professionality I want to develop.’*
***Learning outcomes***
*S5: ‘By reflecting on meaningful experiences, I learned to look more consciously both at things that are going well and at things that I can improve. The individual coaching sessions are a mandatory moment from which to stand still and look back.’*
*S6: ‘The most important thing that I learned during professionalism is to reflect. In the beginning I found it useless and I saw it mainly as a burden, but now it has become a tool to test whether I have made improvements in my skills and learning objectives.’*
*S7: ‘It is amazing that I have learned to speak up if an observation asks for it. Hierarchy was scary for me. I learned to ask questions about observations that puzzled me and to give feedback to my supervisor correctly. The supervisor appreciated my feedback.’*
*S8: ‘Somehow I got the idea that every student should be a leader. However, in patient contacts and discussions with my project team, I realized that followers are needed as well. It is up to you to decide in which role you feel comfortable and use your talents to become a team player.’*
**LPPD – assessment**
*S9: ‘It (red: assessment) just feels more like an obligation than actually working on your development.’*
*S10: ‘I truly did everything (re: the assessment). However, you exaggerate sometimes.’*
*S11: ‘I think that many aspects of professionalism cannot truly be assessed.’*
*S12: ‘You truly have another person (re: the assessor), that you do not know, who has a completely different view. That can sometimes lead to very useful tips.’*
*S13: ‘There are also certain parts of the assessment that I notice, that I have no use of myself. So then I think that this is useless.’*
***(published with permission)***

**Table 4:** Coaches’ quotes concerning the LPPD: program (experiences and learning outcomes) and assessment (interview data 2020–2021)

***LPPD – program***
***Experiences***
*C1: ‘I’m also truly convinced that the professionality program—taking care of yourself, taking care of your relationship with others, taking care of your relationship with your tasks—those are very fundamental characteristics for any professional’*
*C2: ‘… vitality and maintaining motivation for studies are increasingly difficult for many students …. They have a great need for contact with the program and fellow students’*
*C3: ‘To hear students saying: it is a fine LPPD program, not stressful, you know what to do’*
*C4: ‘What an effect individual conversations can have, even they are only for half an hour every 6 weeks’*
*C5: ‘Well, I am jealous, I wish I could have studied like this! It would have helped me a lot, just to discuss like this with people, with coaches, during your study’*
*C6: ‘I noticed a lot of students getting stuck. … I was able to help most students get started again’*
*C7: ‘ Seeing students’ development and noticing that only by asking the right questions can play an important role in that development’*
*C8: ‘In my position as a coach, I am always searching to make a connection with, yes, with becoming a researcher or doctor’*
*C9: ‘Yes, I think [ …] that if you embody what you say, it is more convincing’*
***Learning outcomes***
*C10: ‘In the third year, for most students in the retrospective, comes the realization of how much they have grown and how much they have had (often unnoticed) from LPPD.’*
*C11: ‘To notice that students discuss things, then go out and try them out and learn as a result. This happens regularly in the third year. The student has the necessary baggage behind them from the first two years and deals with it more and more independently.’*
*C12: ‘To see each student continue to grow in their personal area and become aware of talents and pitfalls. Students also appreciate the opportunity to grow further’*
*C13: ‘Individual conversations where students' proverbial 'penny dropped' and they had new insights about themselves, or found support for difficult situations they were experiencing’*
*C14: ‘That I see students push their boundaries by doing things differently, after we have talked about this within professionalism, such as giving feedback to each other’*
**LPPD –assessment**
*C15: ‘As a teacher, I thought it (red: assessment product) was a pretty good overview. It gave me a complete image from which to judge’*
*C16: ‘However, now, sometimes I find that the assessment has so many attachments, and so many things. When I read through it all, I think that I could also have done well with less’*
*C17: ‘I find such a free assignment in which, in my opinion, students write about their development in a more authentic way that they truly have to think about, that is a very good one, that is a very valuable one’*
*C18: ‘The evaluation assignment suits then much better currently’*
***(published with permission)***

### Student perspectives and experiences

From the beginning, the vast majority of the responding students have greatly appreciated the individual coaching and the safe, open environment of a small fixed peer group, in which they feel free to express their feelings and thoughts and to *learn and develop* rather than maintaining a constant focus on achievements (Figs. 3, 4, 5).

Over the undergraduate period of three years, increasing numbers of students have come to appreciate the repetitive attention given to personal awareness, skills training and experimental space. Students expressed that they become more aware of their learning process and come to value the various topics around skills training as well as the connection they develop with the study context (Table 3 S1). They recognize how LPPD stimulates and helps them take responsibility for their learning and develop professionalism in an increasingly complex context (Table 3, S2 and S3). Overall, students report that LPPD stimulates self-directed learning (70%) and the setting of personal learning goals (65%). After their first, second and third academic years, 35%, 58% and 80%, respectively, of the students recognize and value self-reflection skills, as confirmed in in-depth interviews with students (Table 3, S5 and S6). The further into the study they progress, the more that the drive to work on professional development shifts from externally motivated to intrinsically motivated Table 3 S2 and S3).

The obligatory nature of the assessment could cause students to be more concerned with obtaining credits and managing assumed expectations rather than actually learning (Table 3 S9, S10, S13). To obtain greater benefit from the assessment, students suggest an even more free and less-directed assignment to enable student interpretations and additional discourse with the assessor.

### Coach perspectives and experiences

Interviews showed that LPPD is perceived as meaningful for every student (Table 4, C1, C4-7). Coaches add value by providing a combination of group and individual coaching for students over the year (95%). Coaches stimulate peer-assisted learning, observe the interaction, communication skills and professional development of the students (Fig. 4) and discuss these observations in individual coach consultations (90%). According to coaches, LPPD stimulates the professional development of student (Table 4, C10-14), self-directed learning (92%) and personal learning goals (85%). The coach may, at his or her discretion, provide more guidance to a student who needs it, which also provides an opportunity to stimulate above-average students to cross individual boundaries [27].

Coaches provided several insights into the assessment concerning distinctive capability, the stimulation of development and the demand for authenticity and size limits (Table 4, C16, C17). In the actual assignments, they identify differences in personal and professional development among students and thus provide distinction (87%). Expressing oneself through writing needs to and can be learned, as it challenges students to think more deeply. Half of the coaches are convinced that there is sufficient room for student creativity in the program. However, coaches mention some disadvantages, including inequality for students as a result of the vast differences in writing quality among students. Students who find writing to be easy produce socially desirable texts. Although the implemented adaptations have been welcomed (Table 4, C18), half of the coaches (53%) recommend an even more lean assessment protocol.

Nearly every coach reports coaching one or more students with social, psychological or study problems (distress, study load, performance pressure, loss of motivation, perfectionism, fear of failure, mood disorders, choice stress, and loneliness) (Table 4, C2, C6).

## Implications

Elaborate and effective programs, including a framework to stimulate professional development over the years for undergraduate students, lack in literature. Uncontrolled informal learning concerning professional performance and mentoring often results in an undesirable range of consistency and effectiveness for students [10]. Therefore, the aim of our initiative was to develop the necessary framework to prepare new (bio)medical students for the challenges of the future. Evaluation shows that this innovative LPPD helps to facilitate personal and professional growth and identity formation in a safe learning environment that maintains room for diversity, inclusion, discomfort, experiments, and peer-assisted learning. It offers a bridge between knowledge, skills and the experience of applying skills in practice by starting small with a cultural change toward early self-awareness, self-directedness, adaptive expertise and horizontal learning [17]. The transformational journey to become young professionals may take some time, as it requires a learning curve from more or less unconscious incompetence through conscious incompetence toward conscious competence.

The evaluation also provides insights for further improvement. Professionalism tends to be a topic that most students are not familiar with, and it is often referred to as vague, irrelevant or sometimes a waste of time. From literature we know that students have to master skills for which they initially do not see the relevance and need time to come to appreciate and reflect on self-awareness [28]. We envision further improvement through active and challenging work formats and firm alignment with the student’s context to more explicitly make professional development opportunities clear to the student. The cocreated, dynamic and expanding toolbox is one of our solutions for meeting students’ personal preferences and truly connecting with their actual context.

The appropriate assessment of professional development is difficult and may be at odds with the learning process [29]. Professionalism is a complex concept that is not easy to learn or quantify at a performance level. Our written evaluative assessment is meant to be an assessment of preconditions (knowledge about professionalism and the application of trained skills) to become a professional. A lack of familiarity with this type of (essay) assignment from students leads to uncertainty and discomfort. Some students tend to be guided by mostly incorrect assumptions, provide only socially desirable answers or focus on passing the exam, rather than on authentically and personally describing their individual learning process. We need to give students more background and information about the why and how of learning assessments. Another matter of concern is the time-commitment needed for assignments by both students and their assessing coaches. We have to reduce required level of effort to accomplish this. Providing training for assessors and an introduction for students on a regular basis is necessary to both convince students of the power of learning assessments and to guide assessors in giving and applying meaningful, constructive feedback.

The globally identified increase in student mental issues is recognized and explicated in the safe environment of individual coach consultations [30]. To this end, and adding to the literature, this study presents a three year program in which all students are coached. We encourage students to develop strategies based on self-insight to become more resilient and flexible. Besides, we also have to train our coaches. They are overwhelmed by the number of students who suffer from stress, burnout and mental problems. A number of these coaches are themselves healthcare providers. As such, in the case of repeated signs of burnout or mental problems, they are used to giving advice and thinking in terms of solutions. These coaches require active awareness of the teaching role rather than being seen as a health care professional in education. Being a coach is not about giving advice but about asking the student the right questions, thus encouraging them to gain self-insight and solutions that are a personal fit. This role change is not easy for some coaches, and they are asked to refer students for appropriate assistance [9].

It is still uncertain whether students, once they finish their undergraduate curriculum and participate in a Master's internship at a hospital, will be able to continue using the skills they have learned. Based on literature, we know that ambiguity around students’ new roles and expected behavior complicates the socialization process into clinical clerkships [31]. We plan to study the continuous learning journey and the effect of early, explicit attention placed on the professional development of students following their entry into the workplace during clerkships. Students' initial position at the bottom of the medical hierarchy causes regular episodes of moral distress [32]. Our modernly trained students, who have a greater awareness of personal professional identity and attitude, are supervised by professionals who have not been specifically trained themselves in reflective and lifelong learning skills and sometimes even neglect the explicit attention required for reflection or the evaluation of professional skills [33]. Does this gap at the workplace result in the driving of learning by the professional (lifelong learner) or in a loss of trained skills and the demotivation of a Master student? From the perspective of the student, is there a continuous battle between the awareness of the advantages of personal leadership based on reflection with room for diversity and the culture of traditions and hierarchy that is often maintained by senior staff with an emphasis on standardized performance? We are convinced that the investment in the lifelong learning goal by senior staff requires further attention to pave the way for young professionals trained in the competencies important for their future. Our coaches, being professionals and themselves members of a teaching staff, can contribute to the desired culture transformation of the staff by coaching their colleagues. Encouraging students to become professionals does not work if we do not ask for the same skills from our teaching staff [34].

## Conclusion

By imbuing the entire undergraduate curriculum with a comprehensive program regarding personal and professional development, students are enabled to train and apply generic professional skills in a meaningful context. The ‘learning yield’ of LPPD is significant for our institution, and since encouraging lifelong learning and professional development is a generic and cross-curricular topic in education, disciplines other than (bio)medical sciences can benefit from our framework. To translate a framework and program such as this, we recommend that it is clearly embedded within the entire curriculum, including a variety of motivating learning activities, connection with a meaningful context for the student, coaching by dedicated teachers, and enough time for the student to overcome unconsciousness, vagueness and uncomfortableness and become a professional with confidence and resilience. The transformational journey to become young professionals may take students some time but will hopefully lead them to become resilient and flexible professionals.

## Data Availability

The datasets used and/or analyzed during the current study are available from the corresponding author on reasonable request.
